# Editorial: Ethylene Biology and Beyond: Novel Insights in the Ethylene Pathway and Its Interactions

**DOI:** 10.3389/fpls.2020.00248

**Published:** 2020-03-12

**Authors:** Dominique Van Der Straeten, Angelos Kanellis, Panagiotis Kalaitzis, Mondher Bouzayen, Caren Chang, Autar Mattoo, Jin-Song Zhang

**Affiliations:** ^1^Laboratory of Functional Plant Biology, Department of Biology, Ghent University, Ghent, Belgium; ^2^Laboratory of Pharmacognosy, Department of Pharmaceutical Sciences, Aristotle University of Thessaloniki, Thessaloniki, Greece; ^3^Department of Horticultural Genetics and Biotechnology, Mediterranean Agronomic Institute of Chania, Chania, Greece; ^4^INRA Génomique et Biotechnologie des Fruits (GBF), Toulouse, France; ^5^Department of Cell Biology and Molecular Genetics, University of Maryland, College Park, MD, United States; ^6^Beltsville Agricultural Research Center, Agricultural Research Service (USDA), Beltsville, MD, United States; ^7^Institute of Genetics and Developmental Biology, Chinese Academy of Sciences (CAS), Beijing, China

**Keywords:** ethylene, ACC (1-aminocyclopropane-1-carboxylic acid), stress, ACC synthase (ACS), ACC oxidase (ACO), gold standard

This Research Topic presents selected contributions to **Ethylene 2018**, the **XI International Symposium on the Plant Hormone Ethylene**, held in Chania, Greece, on 2nd−6th June 2018, covering exciting new discoveries in the ethylene field.

This issue brings novel insights in ethylene signaling in bacteria, algae, and lower plants as well as evidence supporting a specific role for the ethylene precursor 1-aminocyclopropane-1-carboxylic acid (ACC) in plants.

Ethylene receptors were initially thought to be specific to plants. Interestingly, ethylene receptor homologs have been found in cyanobacteria. It was previously shown that in Synechocystis sp. an ethylene receptor regulates phototaxis and biofilm formation. In this issue, the same group (Allen et al.) demonstrates that another cyanobacterium, the filamentous species Geitlerinema sp. which possesses two ethylene receptor homologs, similarly displays ethylene-dependent alterations in phototaxis, suggesting that such signaling could be prevalent in cyanobacteria. Both species are highly sensitive to ethylene although their ethylene binding characteristics resemble that of plants, thus suggesting signal amplification in cyanobacteria.

In higher plants, subfamily-I of the Arabidopsis receptors (ETR1 and ERS1) and their interactions with downstream players have been extensively studied, while information on subfamily-II (ETR2, ERS2, and EIN4) remains sparse. Here, Berleth et al. demonstrate that ETR2 displays comparable affinities for CTR1 and EIN2 to that previously reported for ETR1, suggesting similar protein-protein interaction-mediated signal transfer for both subfamilies. In addition, the authors show enhanced stability of type-II receptor homomers and type-II:type-I heteromers as compared to type-I homomers, emphasizing the importance of type-II receptors.

Wang and Qiao present a mini-review on the transcriptional regulation of the ethylene response in Arabidopsis. Ethylene signaling involves numerous regulatory steps leading to a diversity of responses in plant growth and development. This review discusses our current understanding of the transcriptional regulation as a major control point in ethylene signaling, focusing on recent insights into the role of chromatin modification in repressing transcription.

Dolgikh et al. summarize recent advances on the molecular mechanisms that underlie EIN3/EIL1-directed ethylene signaling in Arabidopsis, and focus on the role of EIN3/EIL in tuning transcriptional regulation of ethylene response in time and space. Furthermore, they consider the role of EIN3/EIL1-independent regulation of ethylene signaling.

Qin et al. review hormonal crosstalk of ethylene in primary root growth of Arabidopsis and rice. Based on the proposed model, ethylene restricts primary root growth by governing auxin biosynthesis, transport, and signaling through EIN3/EIL1, ERF1, and HB52 interactions in Arabidopsis. ABA and CKs constrain primary root growth by controlling the posttranscriptional regulation of ACS resulting in stimulated ethylene synthesis. GA and ethylene antagonistically regulate stability of DELLA proteins, which act as growth repressors. Low levels of BRs hinder ethylene synthesis by BZR1 and BES1 suppressed ACS gene expression, while high levels of BRs increase ACS stability. Through a different mechanism, ethylene restricts primary root growth in rice, by augmenting auxin and ABA biosynthesis. Understanding light-dependent differences in ethylene synthesis and signaling is essential to expand our insight into the roles of ethylene in growth and development across the plant life cycle.

Harkey et al. performed a meta-analysis of multiple transcriptomic datasets to uncover responses to ethylene that are both light-dependent and light-independent. A set of 139 transcripts with robust and consistent responses to elevated ethylene across root-specific datasets was identified. This “gold standard” group of ethylene-regulated transcripts includes numerous genes encoding proteins that function in ethylene signaling and synthesis. The study further reveals a number of previously uncharacterized factors that may contribute to ethylene response phenotypes.

Plants synthesize ethylene in a two-step reaction starting with the conversion of S-adenosyl-L-methionine (SAM) to 1-aminocyclopropane-1-carboxylic acid (ACC). Vanderstraeten et al. demonstrated that ACC affects early vegetative development independently of ethylene signaling. Using ethylene biosynthesis and signaling inhibitors, as well as mutants, ACC-specific ethylene-independent growth responses in both dark- and light-grown Arabidopsis seedlings were revealed. Hence, researchers employing ACC as an ethylene precursor should be mindful of putative ACC effects confounding ethylene responses in vegetative growth. The exact mechanism underlying the ACC response remains to be identified.

While ACC may play an ethylene-independent role in plant growth and development, the three-membered ring amino-acid is probably also of paramount importance in the balanced co-existence of plants and beneficial micro-organisms around them, surmounting attacks of pathogens.

Though the genome of the pathogenic fungus *Fusarium graminearum*, which infects wheat, seems to lack genes encoding ethylene biosynthetic enzymes, Svoboda et al. identified two ACC deaminase genes, one of which encodes an active ACC deaminase. Considering that ethylene insensitive wheat exhibited higher resistance to *Fusarium* and reduced mycotoxin content, *Fusarium* knockouts of both genes were generated. No differences in the fungal infection and mycotoxin content were detected indicating that ethylene might not affect full *Fusarium* virulence.

Nonaka et al. used ACC deaminase in a Super-Agrobacterium strain to enhance transformation efficiencies of recalcitrant species. Super-Agrobacterium was updated to version 4 by introducing both an ACC deaminase and GABA transaminase gene, encoding enzymes that degrade ACC and GABA, respectively. The use of this modified strain resulted in substantial reduction of the amount of time and labor required for transformations, hence providing a more powerful tool for plant genetic engineering and functional analysis.

While most studies have focused on the conversion of SAM to ACC by ACC synthases, there has been increasing interest in the subsequent step, carried out by ACC oxidases (ACOs). Houben and Van de Poel provide a comprehensive and historical perspective on our understanding of the ACO enzyme, including its discovery, activity, evolution, expression, regulation and potential biotechnological applications.

A thorough analysis of players in ethylene signaling enabled identification of key regulators in fruit development and ripening. Not unexpectedly, translational regulation of ethylene biosynthesis and signaling appears to be fundamental in the control of ethylene effects, besides transcriptional regulation.

Mitalo et al. show that many aspects of kiwifruit ripening can be triggered by ethylene treatment or by low temperature, with the exception of aroma volatile production, a major ripening feature contributing to fruit quality, that is lacking in cold-triggered ripening. The data indicate that the production of aroma compounds is strongly ethylene-dependent and support the notion that in kiwifruit ethylene and low temperature-induced ripening may involve two different regulatory mechanisms.

Chen et al. used a modification of parallel reaction monitoring, a targeted mass spectrometry proteomic method, to study ETR receptor abundance in tomato fruit. Focusing on single peptides of rare proteins, they compared the abundance of ETRs in WT and in the NR mutant. Pearson correlations between mRNA and protein profiles were used as indicators to discriminate the two genotypes and reveal changes over fruit development. It is proposed as an approach to study ETR subfunctionalization across the plant kingdom in development as well as in plant-microorganism interactions.

Regarding the role of ethylene in pigmentation, Khunmuang et al. demonstrate that ethylene-mediated petal color fading in cut Vanda “Sansai Blue” flowers results from degradation of anthocyanidins, cyaniding, and delphinidin, and is not related to the levels of flavonols, such as kaempferol. The endogenous anthocyanin degradation process stimulated by ethylene appears related to increased peroxidase activity but was independent of flower senescence. Espley et al. investigated the incidence of internal browning flesh disorder (IBFD) in high anthocyanin red apples (Malus x domestica). The study, using the highly pigmented “Royal Gala” apple cultivar over-expressing MYB10, revealed that the anthocyanin-related transcription factor is associated with the undesirable fruit disorder. The MYB10 transgenic fruit had a high incidence of IBFD compared to wild type. Interestingly, MYB10-expressor apple had higher expression of ethylene-related genes ACS, ACO, and ERF. Prematurely induced ethylene can advance fruit maturity and, as shown by these authors, may lead to adverse effects on storage of high-anthocyanin fruits.

Hormonal pathways are not functioning as independent routes, but rather contribute to an information web, connecting internal signaling to transduction pathways of external cues. Ethylene is involved in such an intricate network which fine-tunes development from cell and organ specification to senescence and abscission, but also incorporates external signals enabling plasticity in an ever changing environment, and controlling reactions to adverse conditions, such as those encountered as a result of global climate change.

External factors such as wind, hale or snow, and/or growth on uneven terrain, induces tension wood formation in order to reorient and uplift the stem toward its original growth position. Tension wood is characterized by the presence of gelatinous, cellulose-rich (G-)fibers with its microfibrils oriented parallel to the fiber cell axis. Seyfferth et al. demonstrated that ethylene regulates transcriptional responses related to the amount of G-fiber formation and their properties, including chemistry and cellulose microfibril angle, in hybrid aspen. The quantitative and qualitative changes in G-fibers are likely to contribute to uplifting of stems.

Given the importance of ethylene in agricultural applications and the need for sustainable crop production, on the field as well as in post-harvest control of fruits and vegetables, recent research has also focused on improved control of ethylene release and sensitivity, as well as on long-term storage conditions of crop products.

## Author Contributions

All authors listed have made a substantial, direct and intellectual contribution to the work, and approved it for publication.

### Conflict of Interest

The authors declare that the research was conducted in the absence of any commercial or financial relationships that could be construed as a potential conflict of interest.

